# Artificial Intelligence-Assisted Segmentation of a Falx Cerebri Calcification on Cone-Beam Computed Tomography: A Case Report

**DOI:** 10.3390/medicina60122048

**Published:** 2024-12-12

**Authors:** Julien Issa, Alexandre Chidiac, Paul Mozdziak, Bartosz Kempisty, Barbara Dorocka-Bobkowska, Katarzyna Mehr, Marta Dyszkiewicz-Konwińska

**Affiliations:** 1 Chair of Practical Clinical Dentistry, Department of Diagnostics, Poznan University of Medical Sciences, Bukowska 70, 60-812 Poznan, Poland; 2 Doctoral School, Poznań University of Medical Sciences, Bukowska 70, 60-812 Poznan, Poland; 3 Faculty of Medical Sciences, Poznan University of Medical Sciences, Fredry 10, 61-701 Poznan, Poland; 4 Prestage Department of Poultry Sciences, North Carolina State University, Raleigh, NC 27695, USA; 5 Physiology Graduate Program, North Carolina State University, Raleigh, NC 27695, USA; 6 Department of Veterinary Surgery, Institute of Veterinary Medicine, Nicolaus Copernicus University in Torun, 87-100 Torun, Poland; 7 Department of Human Morphology and Embryology, Head of Division of Anatomy, Wrocław Medical University, 50-367 Wrocław, Poland; 8 Center of Assisted Reproduction, Department of Obstetrics and Gynecology, University Hospital and Masaryk University, 601 77 Brno, Czech Republic; 9 Department of Gerostomatology and Pathology of Oral Cavity, Poznan University of Medical Sciences, Bukowska 70, 60-812 Poznan, Poland

**Keywords:** dura mater, algorithms, diagnosis, Cone-Beam Computed Tomography

## Abstract

Intracranial calcifications, particularly within the falx cerebri, serve as crucial diagnostic markers ranging from benign accumulations to signs of severe pathologies. The falx cerebri, a dural fold that separates the cerebral hemispheres, presents challenges in visualization due to its low contrast in standard imaging techniques. Recent advancements in artificial intelligence (AI), particularly in machine learning and deep learning, have significantly transformed radiological diagnostics. This study aims to explore the application of AI in the segmentation and detection of falx cerebri calcifications using Cone-Beam Computed Tomography (CBCT) images through a comprehensive literature review and a detailed case report. The case report presents a 59-year-old patient diagnosed with falx cerebri calcifications whose CBCT images were analyzed using a cloud-based AI platform, demonstrating effectiveness in segmenting these calcifications, although challenges persist in distinguishing these from other cranial structures. A specific search strategy was employed to search electronic databases, yielding four studies exploring AI-based segmentation of the falx cerebri. The review detailed various AI models and their accuracy across different imaging modalities in identifying and segmenting falx cerebri calcifications, also highlighting the gap in publications in this area. In conclusion, further research is needed to improve AI-driven methods for accurately identifying and measuring intracranial calcifications. Advancing AI applications in radiology, particularly for detecting falx cerebri calcifications, could significantly enhance diagnostic precision, support disease monitoring, and inform treatment planning.

## 1. Introduction

Intracranial calcifications are important diagnostic markers that can range from benign accumulations to signs of serious pathological conditions [1]. One specific area of interest is the falx cerebri, a sickle-shaped dural fold that divides the cerebral hemispheres [2]. Positioned centrally within the cranium, it stretches from the crista galli of the ethmoid bone at the front to the internal occipital protuberance at the back [3]. Along its lower edge runs the inferior sagittal sinus, and at the junction where the falx cerebri meets the tentorium cerebelli is found the straight sinus [3,4]. Additionally, the falx cerebri has a biomechanical role in distributing mechanical forces within the brain, which is essential for understanding both trauma and disease progression [2,5].

Calcifications within the falx cerebri can appear as linear, curvilinear, or nodular densities, with varied appearances and clinical implications, suggesting different underlying conditions [6]. The falx cerebri, derived from multipotential mesenchymal cells, has the potential to undergo osteogenic differentiation when subjected to factors such as friction, hemorrhage, or trauma [7]. Calcification within the falx cerebri is generally regarded as a rare occurrence [7,8]. However, the actual prevalence of this phenomenon may be underappreciated [9]. Recent studies employing advanced high-resolution computed tomography (CT) and magnetic resonance imaging (MRI) have revealed that some cases previously identified as dense calcifications on conventional radiography and CT scans might, in fact, consist of a cortical bone outer layer encasing a core of (fatty) bone marrow [9]. This suggests that the frequency of falx cerebri calcification may be higher than previously recognized, necessitating a reassessment of its incidence and underlying mechanisms. These calcifications may be indicative of various pathological processes. For instance, they might indicate the presence of meningiomas, which are common intracranial tumors arising from the meninges that may exhibit calcifications, particularly in their psammomatous type [10]. They might also signal chronic subdural hematomas, characterized by linear or punctate calcifications often linked with repeated hemorrhages and fibrosis [11]. In cases of systemic diseases such as hyperparathyroidism or hypervitaminosis D, diffuse metastatic calcification along the falx cerebri can occur, involving multiple intracranial structures [12]. Moreover, some calcifications are idiopathic, presenting without a discernible cause [13]. These idiopathic calcifications, though typically asymptomatic, still pose a diagnostic challenge, as their presence may complicate the interpretation of neuroimaging and potentially mask or mimic other significant pathologies.

Although some calcifications may be incidental and asymptomatic, necessitating no further action, others warrant careful evaluation due to their potential implications. Despite its clinical and biomechanical importance, the falx cerebri often presents with low contrast in standard imaging techniques like T1-weighted MRI, which challenges its visualization and the assessment of associated pathologies [14].

Cone-Beam Computed Tomography (CBCT) offers high-resolution, three-dimensional digital radiographic images with various fields of view (FOVs), rendering it an important diagnostic tool, particularly for detecting intracranial calcifications [15,16,17]. Utilizing a cone-shaped X-ray beam that rotates around the patient’s head, CBCT captures multiple two-dimensional images which are then reconstructed into three-dimensional scans [16]. Recent research has demonstrated the potential of CBCT in identifying and analyzing intracranial calcifications. A retrospective analysis by Tepe et al. [17] examined CBCT scans of 996 individuals, identifying physiologic calcifications in 49.4% of cases, pineal gland calcifications in 47.6%, bilateral choroid plexus calcifications, petroclinoid calcifications in 22.1%, and falx cerebri calcifications in 6.3%. Another study by Sedghizadeh et al. [18] reported that the majority of the calcifications found on CBCT images in their sample were in the pineal/habenular region (80%), with additional findings in the bilateral choroid plexus region (12%) and the bilateral petroclinoid ligament region (8%). These findings underscore the efficacy of CBCT in identifying and analyzing calcifications in critical regions of the head and neck.

Furthermore, recent developments in artificial intelligence (AI), particularly in machine learning and deep learning, have shown promising results in the field of radiology, including oral and maxillofacial radiology [19]. AI applications have varied from automating the detection of tooth numbering and caries to identifying and analyzing facial asymmetry, periapical lesions, and the inferior alveolar canal on 2D and 3D radiographs [20,21,22,23,24]. AI algorithms have also shown substantial potential in automating the detection and segmentation of calcifications in CBCT images, enabling rapid, accurate analyses such as automatic detection of cervical carotid artery calcification [25]. By leveraging large, annotated datasets, these algorithms can identify subtle patterns and variations in calcifications that may be imperceptible to the human eye. This capability enhances radiological workflows and improves diagnostic accuracy, allowing radiologists to focus on complex interpretation and decision-making tasks.

The application of AI in detecting and segmenting falx cerebri calcifications within CBCT images remains an underexplored area in the current scientific literature. This paper aims to serve as a comprehensive foundation for future research endeavors by systematically highlighting the recent advancements in AI-based segmentation techniques specifically applied to falx cerebri calcifications. It reviews existing studies that have utilized AI to segment the falx cerebri, offering insights into the methodologies and outcomes. Additionally, this paper includes a case report where falx cerebri calcification was successfully segmented using a cloud-based AI algorithm on CBCT images.

## 2. Case Report and AI Application

**Figure 1:**
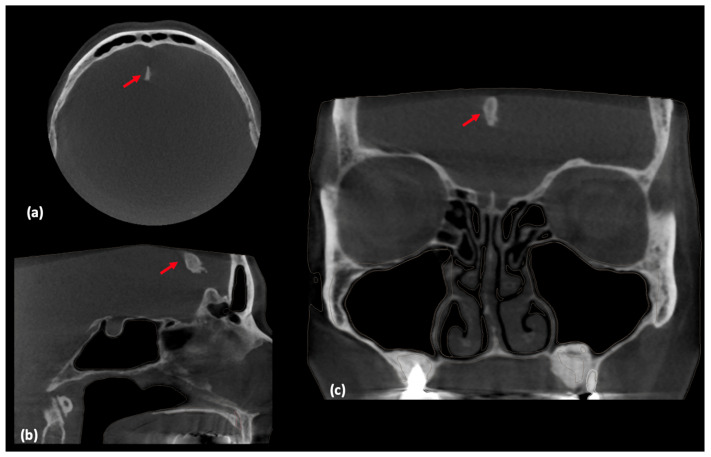
CBCT image view of the falx cerebri calcification (with the red arrow indicating the calcification). (**a**) Axial view. (**b**) Sagittal view. (**c**) Coronal view.

A 59-year-old female patient with a complex medical and dental history who had previously undergone dental implants and a sinus lift two years prior presented with symptoms of right ear blockage, persistent upper respiratory infection lasting one month, and pain in the right maxillary sinus. Additionally, her medical history included the completion of chemotherapy for clear cell ovarian cancer eight years ago (2016). Due to the maxillary sinus pain, she was referred by her dentist for a comprehensive dental and craniofacial evaluation, which was conducted using a Cranex 3D CBCT scanner (Soredex, Helsinki, Finland). The CBCT scan settings were as follows: X-ray tube voltage at 90 kV, X-ray tube current at 10 mA, and a voxel resolution of 0.25 mm. During the examination, a radiolucent mass was accidentally discovered and diagnosed by an oral and maxillofacial radiologist as a calcification of the falx cerebri. This calcification measured 18.6 mm in anteroposterior diameter, 5.8 mm in width, and 8.8 mm in length (Figure 1).

**Figure 2:**
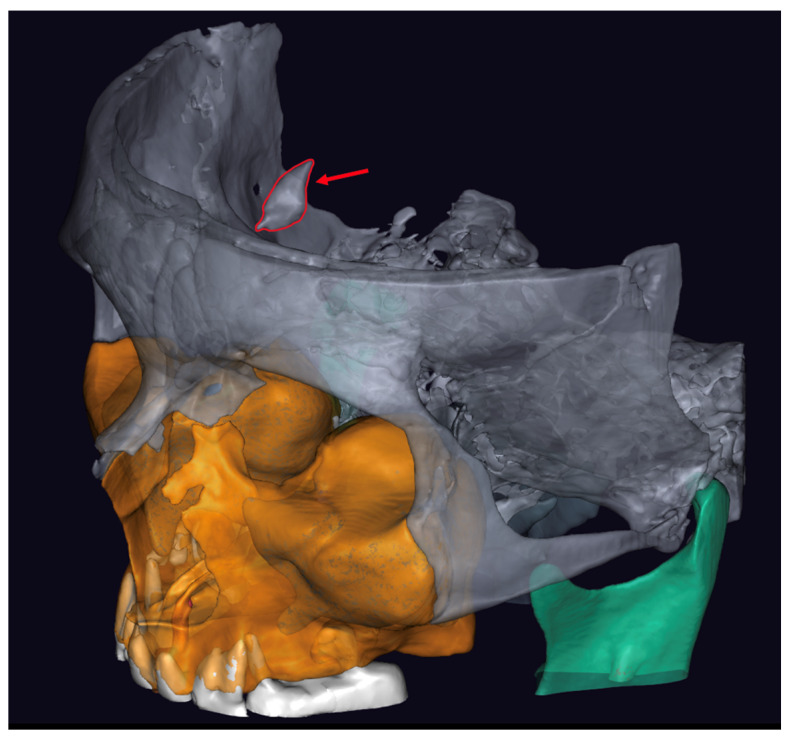
AI-based segmentation of the scanned area (with the red arrow indicating the calcification).

To evaluate the efficacy of AI in segmenting and identifying the falx cerebri calcification, the patient’s CBCT image was uploaded in DICOM (Digital Imaging and Communications in Medicine) format to Diagnocat (Diagnocat LTD, San Francisco, CA, USA), a commercially available cloud-based AI platform for analyzing rental radiographs. Diagnocat (Diagnocat LTD, San Francisco, CA, USA) is designed to store and process dental images utilizing a U-Net-like architecture. Upon processing, the AI tool automatically generated the complete segmentation of the scanned structure, providing the output in standard tessellation language (STL) format for further evaluation (Figure 2).

The falx cerebri was distinctly visible and accurately segmented as part of the cranial structure in the CBCT image analyzed by Diagnocat (Diagnocat LTD, San Francisco, CA, USA). Despite the clarity of the image and the precision of the segmentation, the platform faced challenges with isolating the calcification. Diagnocat (Diagnocat LTD, San Francisco, CA, USA) identified and integrated the calcified area as part of the overall cranial bone, thus preventing a separate analysis of the calcification.

**Figure 3:**
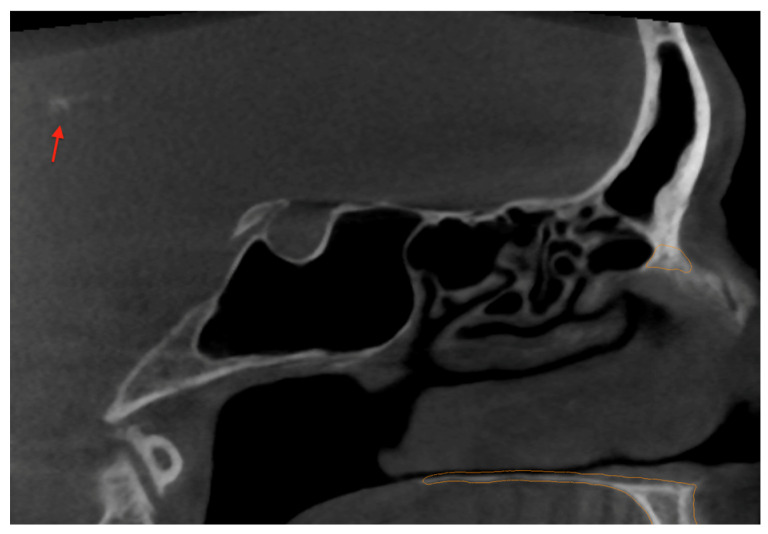
Pineal gland calcification (with the red arrow indicating the calcification).

Furthermore, the patient also exhibited calcifications in the pineal gland (Figure 3), which the Diagnocat (Diagnocat LTD, San Francisco, CA, USA) failed to segment. Given the patient’s history of cancer, it is crucial to emphasize that all calcifications, especially in such high-risk individuals, require vigilant and ongoing monitoring to rule out any potential malignancy or complications.

## 3. Discussion

The integration of AI within dentistry has significantly increased, especially across different dental specialties. However, research into the diagnostic accuracy of AI in various conditions and anatomical areas remains limited. Despite this, the potential benefits of AI, such as increased accuracy, efficiency, and sustainability, underline the importance of continued research in this field.

### 3.1. Literature Review

A comprehensive literature search and analysis were conducted to explore the extent of AI applications in the detection and segmentation of falx cerebri calcifications using various imaging modalities. This search was carried out across several databases using the following Medical Subject Heading (MeSH) keywords: “Falx Cerebri” OR “Cerebral Falx “combined with “artificial intelligence” OR “AI” OR “deep learning” OR “machine learning”. This search strategy was designed to capture a wide range of studies related to the application of AI in the segmentation and detection of the falx cerebri. As a result, four significant studies were identified that have trained and tested AI-based tools specifically for the segmentation and detection of the falx cerebri across different imaging modalities (Table 1).

The four reviewed studies employed computed tomography (CT) images for the training and validation of their respective algorithms [26,28,29], with one study additionally utilizing MRI [27]. Geographically, the research is distributed across three continents, with two studies originating from Europe [26,29], one from the United States of America [27], and one from Asia [28]. Among these publications, three are original articles [27,28,29], while one presents its findings as a conference paper [26].

Grigaitis et al. [26] developed a fuzzy logic algorithm for detecting the falx cerebri curve in CT scans but did not report accuracy and dice similarity coefficients for the method. Shusharina et al. [27] achieved a dice similarity coefficient of 0.97 with 2 mm tolerance, demonstrating the high precision of their U-Net neural network for falx cerebri segmentation in CT and MRI scans of 206 patients. Angkurawaranon et al. [28] documented an 89% accuracy rate for their DeepMedic neural network in localizing and classifying traumatic brain hemorrhage areas, including the falx cerebri, in 300 CT scans. Lastly, Puzio et al. [29] reported a dice coefficient of 0.76 for their U-Net neural network in segmenting the brain compartments in CT scans of 274 patients. Among these studies, only Grigaitis et al. [26] focused solely on the segmentation of the falx cerebri.

The review of the literature indicates that the four analyzed studies predominantly used CT images for the training and development of their algorithms, with none utilizing CBCT as a potential imaging modality. From the first publication in 2007 [26] to the most recent in 2024 [29], only four studies over 17 years have addressed this topic. This highlights a significant research gap and underscores the need for more studies exploring the potential applications of AI in this field, which could lead to improved treatment planning and enhanced patient quality of life. Expanding research to include CBCT and other imaging modalities could further advance the capabilities of AI in medical imaging and provide more comprehensive diagnostic tools.

### 3.2. Intracranial Calcification Detection on CBCT

One area where AI applications are notably lacking is in the detection and segmentation of head and neck calcifications in CBCT scans. These calcifications often go unnoticed, particularly in evaluations performed by dentists focused mainly on dental issues. The shortage of oral and maxillofacial radiologists in many regions across the globe further exacerbates this issue, leading to significant under-reporting of calcifications in CBCT scans [30]. The lack of standardized education on interpreting these scans adds to these challenges [24,30,31], highlighting the potential of AI to identify life-threatening calcifications, thereby significantly benefiting patient health.

Segmenting calcifications in CBCT images presents various difficulties. These images can sometimes contain noise and artifacts arising from various sources, such as scatter radiation, beam hardening, and patient movement during the scanning process, which can affect the visibility of calcifications [31]. The contrast between calcifications and surrounding tissues might be low, particularly if the calcifications are small or located in dense areas, making it challenging for algorithms to detect them. These calcifications can vary greatly in size, shape, and appearance, necessitating robust algorithms capable of accurate detection despite these variances. Additionally, calcifications may overlap with other anatomical structures, making it even more difficult to distinguish them from surrounding tissues, especially in complex regions of the head and neck.

Despite these challenges, AI’s role in identifying specific types of calcifications, like cervical carotid artery calcifications (CACs), has been demonstrated. Ajami et al. [25] utilized a convolutional neural network (CNN) algorithm that successfully localized CACs in CBCT images, achieving a sensitivity of 94.2% and a specificity of 96.5%. This achievement underscores the potential of AI in this domain; however, it is important to note that other types of calcifications remain underexplored. The limited scope of current AI applications highlights the need for further research and development in this field.

In this case report, although Diagnocat (Diagnocat LTD, San Francisco, CA, USA), was able to segment falx cerebri calcifications, it failed to classify them as distinct entities and lacked alert functionalities for such findings in CBCT images, although these features exist for panoramic and periapical X-rays. The algorithm also encountered difficulties in identifying and segmenting smaller calcifications, such as those found in the pineal gland, which are visible on CBCT images.

Deep learning, particularly convolutional neural networks (CNNs), has shown remarkable success in various medical image analysis tasks [32]. Continued advancements in deep learning architectures, training strategies, and optimization algorithms are likely to improve the accuracy and efficiency of intracranial calcification detection algorithms. Integrating information from multiple imaging modalities, such as CT, MRI, and positron emission tomography (PET) scans, could enhance the sensitivity and specificity of calcification detection algorithms. Multi-modal fusion approaches, combined with advanced deep learning models, have the potential to leverage complementary information from different imaging modalities, thereby significantly enhancing overall performance.

Beyond simple detection, future algorithms may focus on quantifying the characteristics of intracranial calcifications, such as size, shape, distribution, and temporal changes. This quantitative analysis could offer valuable insights for risk assessment, disease progression monitoring, and treatment strategy planning. Automatic calcification detection algorithms can be integrated into clinical decision support systems to assist radiologists in interpreting medical images efficiently and accurately. These systems can provide real-time feedback, highlight regions of interest, and generate quantitative reports to aid in clinical decision-making.

As healthcare moves toward personalized medicine, there is growing interest in tailoring medical imaging analysis algorithms to individual patient characteristics and disease profiles [33]. Future algorithms may leverage patient-specific data, including genetic information, medical history, and treatment responses, to enhance the accuracy and relevance of intracranial calcification detection. By incorporating these personalized elements, AI algorithms could offer even greater precision in detecting and managing calcifications, ultimately improving patient outcomes.

## 4. Conclusions

The cloud-based AI software implemented in this case study demonstrated promising results in segmenting falx cerebri calcifications. Despite these promising results, the literature review revealed a significant gap in research: only four studies have addressed the application of AI in the segmentation and identification of falx cerebri, and notably, none have specifically tested AI for falx cerebri segmentation using CBCT images. This emphasizes the need for further research to explore AI-based segmentation techniques for falx cerebri calcifications across various imaging modalities, including CBCT, to better understand their potential clinical applications.

## Figures and Tables

**Table 1:** Studies testing the application of AI in detecting and segmenting the falx cerebri on various image modalities. N/A: Not available.

Author, Study Location, and Year of Publication	Type	Aim	Sample Size (Patients)	Imaging Modality	Algorithm	Accuracy	Dice Similarity Coefficients
Grigaitis et al., Lithuania, 2007 [26]	Conference paper	Developing a plain detection algorithm that detects the falx cerebri curve on each human brain computed tomography slice.	91	Computed tomography (CT)	Fuzzy logic	N/A	N/A
Shusharina et al., USA, 2020 [27]	Original article	Developing a method for automated expansion of the gross tumor volume and applying it to the delineation of the clinical target volume for highly infiltrative tumors.	206	Computed tomography (CT) and magnetic resonance imaging (MRI)	U-Net (convolutional neural network)	N/A	0.97 (falx cerebi with 2 mm tolerance)
Angkurawaranon et al., Thailand, 2023 [28]	Original article	Testing the diagnostic performance of a deep learning model in the localization and classification of traumatic intracranial hemorrhages compared to radiology, emergency medicine, and neurosurgery residents.	300	Computed tomography (CT)	DeepMedic (convolutional neural network)	89%	N/A
Puzio et al., Poland, 2024 [29]	Original article	Developing an algorithm to quantify the mass effect requiring neurosurgical treatment on emergency head CT scans.	274	Computed tomography (CT)	U-Net (convolutional neural network)	N/A	0.76

## Data Availability

The raw data supporting the conclusions of this article will be made available by the authors on request.
